# Distribution of Phlebotomine Sand Flies (Diptera: Psychodidae) in the Lombardy Region, Northern Italy

**DOI:** 10.3390/insects13050463

**Published:** 2022-05-16

**Authors:** Francesco Defilippo, Maya Carrera, Davide Lelli, Sabrina Canziani, Ana Moreno, Enrica Sozzi, Giovanni Manarolla, Mario Chiari, Farioli Marco, Monica Pierangela Cerioli, Antonio Lavazza

**Affiliations:** 1 Istituto Zooprofilattico Sperimentale della Lombardia ed Emilia-Romagnia, Via Bianchi 9, 24124 Brescia, Italy; maya.carrera@izsler.it (M.C.); davide.lelli@izsler.it (D.L.); sabrina.canziani@izsler.it (S.C.); anamaria.morenomartin@izsler.it (A.M.); enrica.sozzi@izsler.it (E.S.); monicapierangela.cerioli@izsler.it (M.P.C.); antonio.lavazza@izsler.it (A.L.); 2 Welfare Department, Lombardy Region, Piazza Città di Lombardia 1, 20124 Milan, Italy; giovanni_manarolla@regione.lombardia.it (G.M.); mario_chiari@regione.lombardia.it (M.C.); marco_farioli@regione.lombardia.it (F.M.)

**Keywords:** sand flies, *Leishmania* surveillance, *Phlebotomus ariasi*, Italy

## Abstract

**Simple Summary:**

Pathogens transmitted to humans and animals by Phlebotomines are relatively neglected, as they cause infectious diseases which represent an underestimated burden in most European countries. Several sand fly species are competent vectors of Leishmaniasis, an endemic disease that has spread widely throughout the Mediterranean region in conjunction with sand flies’ movements. In the Lombardy region, information on sand flies is poor and/or outdated. Therefore, the present study was undertaken to preliminarily ascertain the species composition, distribution, and diversity in representative Lombardy localities. The sampling took advantage of regional surveillance plans namely, West Nile virus and leishmaniasis monitoring plans. A focused sampling was also performed in areas identified as favorable for vector presence. Sampling was conducted using CO_2_–CDC traps conducted every two and/or three weeks. From trapping for the West Nile monitoring plan, 21 out of 44 capture sites were positive for sand flies, while for the leishmaniasis monitoring plan, 11 out of the 40 trapping sites detected sand flies’ presence. Specimen identification was conducted by identifying morphological features. *Phlebotomus perniciosus* was the most abundant species (87.76% of specimens collected). Adequate and well-structured monitoring of sand fly populations is essential to provide information about distribution patterns of vector species present in defined geographical areas, as they could enhance pathogen circulation.

**Abstract:**

This study investigated the species composition and density of sand flies in the Lombardy region (Northern Italy). Sand flies were collected using CDC traps baited with CO_2_ (CO_2_–CDC traps) between June and August 2021. A total of 670 sand flies were collected. The specimens were identified as seven species belonging to two genera, *Phlebotomus* and *Sergentomyia*, namely, *S. minuta*, *Ph. perniciosus*, *Ph. perfiliewii*, *Ph. neglectus*, *Ph. mascitti*, *Ph. papatasi*, and *Ph. ariasi*. *Phlebotomus perniciosus* was the most abundant species (87.76%), followed by *Ph. perfiliewii* (7.31%), *Ph. neglectus* (3.13%), *S. minuta* (0.75%), *Ph. mascitti* (0.6%), *Ph. papatasi* (0.3%), and *Ph. ariasi*, for which only one specimen was identified. Among these identified species, five are considered vectors of *Leishmania*, which causes cutaneous and visceral leishmaniasis. As vector presence increases the risk of vector-borne leishmaniasis, these results suggest that Northern Italy could be a potential area of pathogen circulation over the next few years. These preliminary results suggest that the risk of borne leishmaniasis is high in this region of Northern Italy. Monitoring the distribution of sand fly species in areas suitable for their persistence is important for control programs aimed at reducing the risk of leishmaniasis infection.

## 1. Introduction

Phlebotomine sand flies (Diptera: Psychodidae) are hematophagous insects that can act as vectors of different pathogens. The agents transmitted to humans and animals by phlebotomine sand flies are relatively neglected in research, as they cause infectious diseases which are poorly reported but represent an underestimated burden in most European countries [1]. In the last few decades, these diseases have been emerging in southern Europe, and they seem likely to continue to spread in the future given the projected expansion of areas with even more favorable climatic conditions resulting from climate change [2]. Among sand fly-borne pathogens, the most relevant are viruses belonging to the *Phlebovirus* genus (e.g., the Toscana virus), *Bartonella bacilliformis*, and protozoa of the genus *Leishmania* [3], commonly transmitted from an animal reservoir to humans [4]. In addition, there is a clear correlation between the presence of vectors in endemic areas and the epidemiology of these neglected diseases [3].

In Europe, several regions host endemic Leishmania species, which cause mostly visceral (VL) and cutaneous (CL) leishmaniasis in humans as well as Canine Leishmaniasis (CanL) in canids. Four species can be found in Europe: *L. tropica, L. major*, and the *L. donovani complex* species, including *L. infantum* and *L. donovani* s.s. [4,5]. *Leishmania tropica* and *L. major* were sporadically reported, the first in Cyprus and Serbia and the latter in Georgia. *Leishmania donovani* s.s. was found only in Cyprus. Thus, *L. infantum* is the predominant species in Europe where there are several specific animal reservoirs, such as rodents, marsupials, edentates, monkeys, domestic dogs and wild canids [5,6].

Since the 1990s, the incidence of human visceral and cutaneous leishmaniasis has been increasing in Italy, with new foci detected within classical endemic areas (regions and islands in the southern peninsula) and in northern regions previously regarded as non-endemic [4]. Sporadic cases of CL are often reported, even though the pathology is benign and rarely requires hospitalization [7]. However, the exact prevalence of cutaneous and visceral leishmaniasis is largely unknown, as underdiagnosis and underreporting are common [8,9]. The severe form of the disease, visceral leishmaniasis (VL), is well defined instead, with several studies monitoring its distribution; in fact, since the beginning of 2000 more than two hundred cases per year have been registered in Italy [7].

The spread of vectors in areas regarded as non-endemic in the last few decades [10] made Leishmaniasis a public health concern [11].

Strategies for vector control are fundamental for preventing leishmaniasis in animal reservoirs, considering their crucial role in the Leishmania transmission cycle. These strategies should be directed to achieve three main goals: (i) the implementation of monitoring programs, which are necessary for evaluating the presence/distribution of phlebotomines, and the efficacy of measures adopted for their control; (ii) the vector maintenance using insecticides and environmental control; (iii) the adoption of measures aimed at preventing contact between phlebotomines and animal reservoirs [12].

In this context, a regional plan for monitoring leishmaniasis was initiated in 2021 in the Lombardy region (Northern Italy) with the aim of acquiring data on vector distribution through systematic entomological monitoring. Traps were located inside or close to sanitary kennels, to detect phlebotomines in the proximity of their major reservoir, i.e., dogs. Data were implemented through an ongoing additional monitoring program, i.e., the West Nile disease monitoring plan that involves the placement of traps in farms or agricultural fields.

Herein, we report the results of the collection and identification of phlebotomine sand flies in the Lombardy region through entomological surveillance.

## 2. Materials and Methods

### 2.1. Study Area

Lombardy has a relatively simple geographical structure. The northern part of the region is essentially mountainous (more than 40% of the regional territory is occupied by mountains), while the remaining southern half consists of lowlands (with more than 47% of the region occupied by plains) that stretch across the central part of the Po Valley and not through the southwestern part, the Oltrepò Pavese, where the hills of the Apennines are located. A strong anthropic modification, with the abundant presence of industrial and urban settlements, characterizes the surveyed area. The rural part of the territory is connoted by intensive agriculture and animal husbandry.

The climate of Lombardy is classified as continental, while the mountainous areas above 1500 m show characteristics typical of high Alpine mountains. However, the climatic characteristics of Lombardy can vary considerably, even among areas that are not very distant from one another, owing to the presence of hills, exposure to prevailing winds, and the presence of large lake basins, which can bring Mediterranean characteristics to the climate of immediately surrounding areas. Temperatures vary depending on the exposure and altitude of the area. Summers are hot, with values easily exceeding 30 °C. During heat waves, temperatures can exceed 35 °C in inland basins and in the Po Valley.

### 2.2. Sand Fly Collection and Identification

The present study combines sampling according to two different monitoring plans (leishmaniasis and West Nile Virus). The two plans (for WND and Leishmania) were not deliberately combined; on the contrary, efforts were made to keep them as distinct as possible, so much so that trapping was not only carried out every other week but also at different sites. Thus, the sampling frame allowed a degree of overlap in time as periods of abundance of both sand flies and mosquitoes coincide, at least in the climatic and environmental conditions of the Po Valley in the Lombardy region [13].

The WND surveillance program has been performed in Lombardy since 2010 in accordance with the national plan of the Ministry of Health for WND human surveillance that integrated human and veterinary surveillance. The plan conducted in Lombardy [14] and Emilia Romagna [15] is based on entomological and veterinary surveillance (birds and horses) and is carried out during the period of major presence and distribution of mosquitoes in the Po Valley, which begins in June and lasts until the end of September.

A leishmaniasis monitoring plan was applied for the first time in 2021: 40 public kennels distributed throughout the whole region in peridomestic environment were put under surveillance. WNV surveillance plan was implemented in Lombardy starting in 2014 with the aim of detecting virus circulation in mosquitoes. Surveillance focused on rural areas and farms. During the WNV monitoring plan, other blood-sucking dipterans were collected, including phlebotomine sand flies. For this reason, we decided to combine the data of both surveillance plans.

The extensive field sampling to monitor sand flies was performed during the sand fly activity seasons, which, according to the literature data [13], lasts from the beginning of June to the beginning of October 2021 in the whole territory of Lombardy [13,16].

Trapping sites in the proximity of dog shelters and sanitary kennels were selected for the leishmaniasis monitoring plan (for a total of 40 trapping sites). A trap was placed at each site. The traps for the West Nile virus plan were set in farms or agricultural fields (for a total of 44 trapping sites). To define the position of West Nile traps the area was divided into a grid with a length of 20 km. The traps were placed ~20 km apart. A trap for collecting host-seeking mosquitoes was placed in each geographical unit. Sampling areas were located at altitudes between 10 and 567 m above sea level (a.s.l.). Additional focused sampling was performed in Collebeato, a village identified as favorable for the persistence of sand flies, considering the temperature (a lowest mean temperature of 18 °C), altitude (363 m a.s.l.), and environmental conditions (flora composed of bushes and isolated edifices).

The collections were performed using modified CDC traps baited with CO_2_ (CO_2_–CDC traps) [17].

CDC traps were filled with dry ice pellets as a source of carbon dioxide in order to attract hematophagous insects and powered by a 12 V battery [17]. According to Hoel et al. [18], the CO_2_-baited traps catch higher numbers of sand flies than light traps without CO_2_.

All traps were georeferenced and set at night for operation from roughly 5:00 p.m. to 9:00 a.m. Each site regularly included in the two surveillance plans was sampled every fortnight and every twenty-one days for the West Nile and leishmania plans, respectively.

In order to identify the sand fly species, the head and the rear of the abdomen of all the sand flies collected were clarified using chloral hydrate and acetic acid and mounted in Hoyer’s solution on permanent microscope slides. Females were identified by examining the morphology of the pharyngeal armatures and spermathecae. For males, the external genitalia were examined and the number of coxite hairs were counted according to the morphology-based keys described in Dantas-Torres et al. [19].

### 2.3. Statistical Analysis

Since the number of phlebotomine sand flies other than *Ph. perniciosus* was very low, this species was only considered for statistical analysis. In order to improve the data evaluation and better understand the ecological diversity of the sand fly populations, specific ecological indexes, calculated over the total number of sand flies identified during the season at three sites located at different altitudes (A = 10–200 m a.s.l., B = 200–300 m a.s.l., and C = over 300 m a.s.l.), were included in the study. In particular, the following dominance and diversity indexes were employed:

(i) Simpson’s diversity index (λ = 1 − ∑ pi2), where pi = ni/N (ni = the number of individuals in the taxon ‘i’ and *n* = the total number of individuals) [20];
(ii) The Shannon diversity index (H’ = −[∑ (pi lnpi)]), which is commonly used to characterize the diversity of species in a community through consideration of both the abundance and the uniformity of the species present [20,21];
(iii) The Berger–Parker dominance index (D) defines the level of dominance within different collection sites [22].

The Chi-square test was used to compare the presence of *Ph. perniciosus* at different altitudes. The analysis was conducted using MedCalc statistical software version 13.1.0 (MedCalc Software bvba, Ostend, Belgium). *p* < 0.05 was chosen as the level for statistically significant differences based on comparisons.

## 3. Results

**Figure 1:**
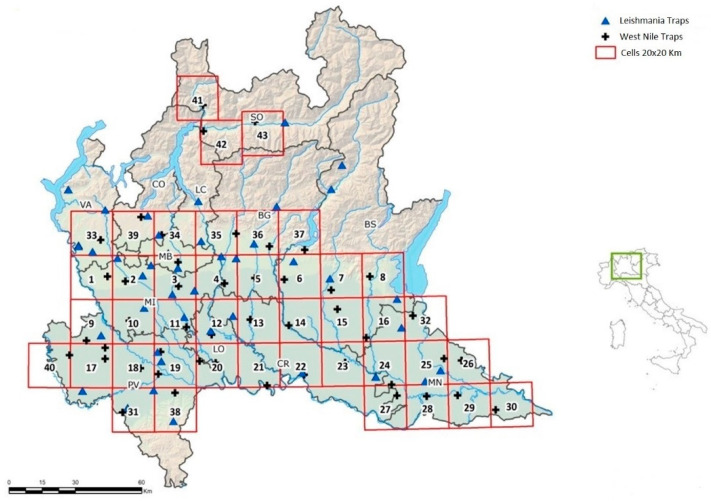
Geographical location of CO_2_–CDC traps at the collection sites. The collection sites are represented with blue triangles and black crosses for leishmania and WNV surveillance, respectively.

The sand flies were collected from 1 June to 1 October in different areas of the Lombardy region (Figure 1). Positive samplings were observed from 15 June to 30 August 2021.

There were 66 days of capture throughout the breeding season when the daily mean temperature ranged from 20.3 to 29.6 °C. For the West Nile monitoring plan, CO_2_–CDC traps for 21 out of 44 (47.73%) capture sites were positive for sand flies, while for the leishmaniasis monitoring plan, 11 out of the 40 (27.5%) trapping sites all over Lombardy were positive for sand fly presence.

A total of 670 sand flies were captured, inclusive of male and female sand flies. Regarding species identification, *Ph. perniciosus* was the most abundant species (87.76%), followed by *Ph. perfiliewii* (7.31%) and *Ph. neglectus* (3.13%) (Table 1). For each collection site the number of phlebotomine sand flies sampled and the altitude of recovery are reported in Supplementary Materials: Table S1.

The number of specimens of each species collected in the three different sites typology (WN surveillance, *Leishmania* surveillance and Collebeato Village) is reported in Table 2.

**Figure 2:**
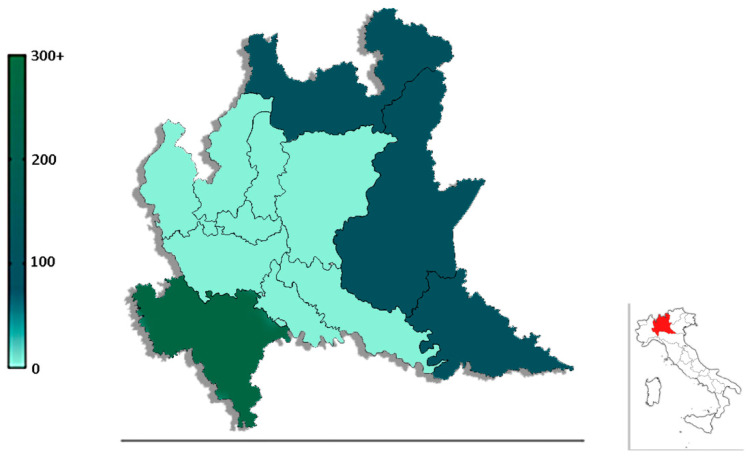
Species abundance of sand flies collected in each province of the Lombardy region.

The trapping sites on the northern and eastern sides of the region had the greatest richness, with seven different species (*Ph. perniciosus, Ph. perfiliewii*, *Ph. neglectus, S. minuta, Ph. mascitti, Ph. ariasi*, and *Ph. papatasi*) being recorded (Figure 2).

Sand flies were collected at different altitudes ranging from 10 m a.s.l. (Poggio Rusco) to 567 m a.s.l. (Montagna in Valtellina).

*Ph. perniciosus* and *Ph. perfiliewii* were distributed over several altitudes, with both presenting within a range of a maximum of 415 m a.s.l. and a minimum of 17 m a.s.l. *Ph. neglectus* presented at a median altitude of 316 m a.s.l., and its range was between 161 and 385 m a.s.l. *Ph. mascitti* was found at a similar range, with a maximum altitude of 385 m a.s.l. and a minimum of 192 m a.s.l. as well as a median value of 374 m a.s.l. *S. minuta* and *Ph. papatasi* were found at median altitude values of 53.5 and 121 m a.s.l., respectively. They were collected at the lowest altitudes of all collected species.

**Figure 3:**
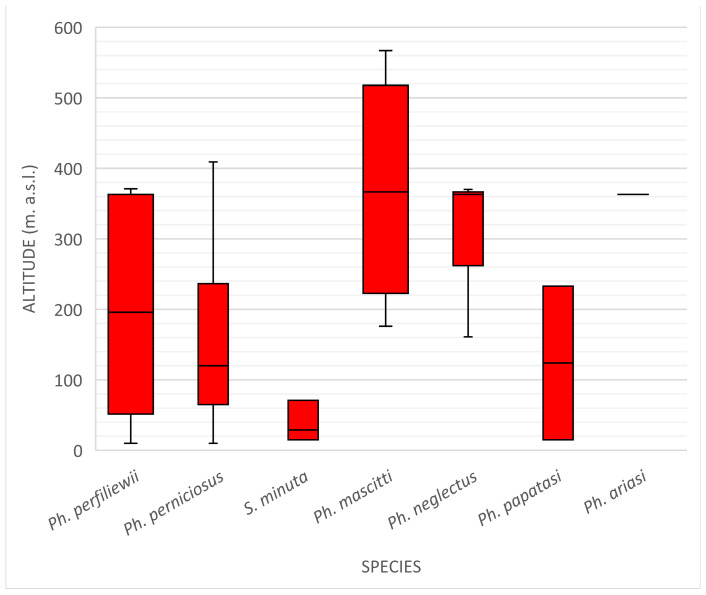
Box diagram describing the altitude range for each sand flies species. Only one specimen of *Ph. ariasi* was collected, so the altitude is shown in the graph as a line at 363 m a.s.l.

The box diagram in Figure 3 shows the presence of each Phlebotominae species in the function of the altitudinal gradient.

Some important diversity indices were calculated, including species richness, Shannon index, Simpson index, and Berger–Parker dominance index. The results of these calculations are shown in Table 3.

The samples of *Ph. perniciosus* from the three sites considered were found to be significantly different (*p* < 0.0001).

## 4. Discussion

In this preliminary study, we report on the presence of phlebotomine sand flies in the Lombardy region during the summer of 2021 according to the surveillance plans for leishmaniasis and WNV.

In our study, a uniform trapping method was employed, as required, for the comparison of fly abundance [23]. Therefore, CO_2_–CDC traps were used because they are considered more effective than other traps (i.e., sticky traps or CDC light traps) for collecting sand flies [24].

The samples showed a different distribution of species abundance within the Lombardy region, with a greater abundance captured in the northeastern than the southwestern area. Indeed, the greatest abundance of sand flies was found in areas confined within districts of the neighboring Veneto region, which is consistent with previous findings [13,25]. An increase in the diversity distribution area of sand flies was observed in this region, and *Ph. perniciosus* and *Ph. perfiliewii* were collected from the whole regional territory. These data may suggest the spread of sand flies from confined areas recognized as endemic foci for leishmaniasis (Veneto from the east and Piedmont from the west) and therefore characterized by associated with the abundance of the vector [7].

In this study, *Ph. perniciosus*, i.e., the most abundant phlebotomine sand fly species in Italy and a proven vector of VL and CL [26,27], was found to be the dominant species. Its presence was confirmed in the study area, even in lowlands (17 m a.s.l.), suggesting an adaptation to environments characterized by low altitude sites with a continental climate and peridomestic environment [13,28].

Among the other identified species, particularly interesting results were obtained from the sampling of *Ph. neglectus, Ph. mascitti,* and *Ph. ariasi*, each of which represents a potential vector for Leishmania diffusion.

*Ph. neglectus* is considered one of the most competent vectors of *L. infantum* [29]. It was found in the Apennine peninsula and in the Eastern Mediterranean area. The results confirm previous observations on the presence of *Ph. neglectus* in Northern Italy and suggest that Italy represents the western limit from Northern Italy to the southern coast of the Mediterranean Sea [6,30,31].

*Ph. mascitti* is a suspected vector of *L. infantum* [6,32]. However, its vector competence has still not been experimentally verified, although the repeated signals of leishmaniasis cases in Germany and Austria [33,34] may indicate the possible role of *Ph. mascitti* in *L. infantum* transmission [27,35,36].

*Ph. ariasi* is confined to the Mediterranean areas of Western Europe and North Africa. This species was successively collected in Spain, France, Algeria, Morocco, Portugal, Italy, and Tunisia [37]. Concerning its distribution in Italy, *Ph. ariasi* has been exclusively reported in two regions since 1964, namely Piedmont and Liguria [19,38]; this is therefore the first time that it was found in the Lombardy region. This species shares its habitat with *Ph. perniciosus* under sympatric conditions and, thus, they are likely able to act together as vectors in a single outbreak [38]. In Italy, *Ph. ariasi* was identified at elevations ranging from 90 to 1060 m a.s.l., with the majority found at f 500–600 m a.s.l. [19]. *Phlebotomus ariasi* is also a likely phlebovirus vector [39].

Our findings also suggest the adaptation of sand flies to environments with different altitudes and to continental climates [40]. This strong influence of altitude was described by Prudhomme et al. [41]. A strict relationship between sand flies and the environment can lead to the formation of ecotypes in the phlebotomine population. Therefore, it will be necessary to consider sand fly populations on a small and specific scale to determine their ecology and their impact on *Leishmania* transmission.

In the study area, there were differences in species richness and diversity indices within the three altitudinal ranges considered. The highest values of the Shannon diversity index were at site C with higher altitudes, and its values were higher at site A than at site B. A community is said to have a high species diversity if many species are present and all are nearly equally abundant. In all the sites in the study area, one species (*Ph. perniciosus*) was dominant, as indicated by the Berger–Parker dominance index; evenness was therefore lower, and the species diversity was not high. Diversity indices may differ over a number of years, and sites should therefore be compared over several sampling seasons [42]. Unfortunately, at present, there are no studies on the biodiversity of sand flies in Italy. In this context, our observations should be repeated over a number of years in order to increase the value of phlebotomine community surveillance within the *Leishmania* surveillance area.

Some points of criticism may of course be mentioned, such as that our sampling sites were not specifically located in areas suitable for these insects and that our data do not show the seasonal activity of sand flies. This study took advantage of the execution of the Leishmania monitoring plan, which permitted us to preliminarily know the distribution of the sand fly, and the obtained results provide important baseline data on sand fly species presence and abundance trends. Indeed, they suggest, for the future, the planning of specific entomological surveillance focusing on the detection of sand flies to obtain an even more realistic picture of their distribution in the area. In fact, only by having the map of the distribution of the competent and potential vectors of *Leishmania* spp., will it be possible to determine the real risk of occurrence and diffusion of the disease in humans and animals in those areas, such as Lombardy, which were still considered free from the infection.

## 5. Conclusions

Our sampling activity in the Lombardy region enabled us to highlight *Ph. perniciosus*, representing approximately 90% of the specimens collected, as a potential Leishmania vector. The presence of several species collected in low-altitude areas, such as *Ph. perfiliewii* and *Ph. Neglectus,* as well as *Ph. mascitti,* could contribute to pathogen circulation, as documented in the onset of epidemic outbreaks of leishmaniasis and other sand fly transmitted diseases in areas previously considered non-endemic [43]. Continuous monitoring of population dynamics is necessary for areas in which elements of the transmission cycle are found.

Focused sampling enabled us to identify habitats favorable species not previously registered in the Lombardy region, such as *Ph. ariasi*. Further investigations will be conducted to increase knowledge about the actual distribution of *Ph.ariasi* and other species.

In conclusion, the characteristic distribution of sand flies, which was identified in this study as typically discontinuous, should be more precisely mapped in the different sampling areas through future more detailed studies of the territory. Therefore, an adequate system for the monitoring and surveillance of the sand fly population may provide information about the main vector species and their distribution over the territory during periods of activity as well as help to define pathogen circulation in a defined geographical area.

## Figures and Tables

**Table 1:** Sand fly species identified in Lombardy from 1 June to 1 October 2021.

Species	Number of Specimens Identified	%
*Ph. ariasi*	1	0.15
*Ph. mascitti*	4	0.60
*Ph. neglectus*	21	3.13
*Ph. papatasi*	2	0.30
*Ph. perfiliewii*	49	7.31
*Ph. perniciosus*	588	87.76
*S. minuta*	5	0.75
Total	670	100

**Table 2:** Species and number of Phlebotomine sand flies collected per site typology.

Species	Number of Specimens in WND Sites	Number of Specimens in Leishmania Sites	Number of Specimens in Collebeato Village
*Ph. perniciosus*	164	340	84
*Ph. perfiliewi*	39	5	5
*Ph. neglectus*	2	0	19
*Ph. mascittii*	1	1	2
*Ph. papatasi*	2	0	0
*S. minuta*	4	1	0
*Ph. ariasi*	0	0	1

**Table 3:** Diversity indices of the sand flies from the collection sites at three different altitudes in the Lombardy region in 2021 * (A = 10–200 m a.s.l., B = 200–300 m a.s.l., and C = over 300 m a.s.l.).

Index	Computation	Collection Sites *		
		A	B	C
Specimens	Total number of specimens	163	392	115
Species richness (S)	Number of species	6	3	5
Simpson index (λ)	λ = 1 − ∑ pi^2^	0.4119	0.0253	0.5633
Shannon index (H’)	H = −[Σ (pi lnpi)]	0.2035	0.0128	0.3618
Berger–Parker dominance index (D)	D = ni/N	0.7669	0.9872	0.6609

## Data Availability

Data sharing not applicable.

## Supplementary Materials

The following supporting information can be downloaded at https://www.mdpi.com/article/10.3390/insects13050463/s1: Table S1: sand flies collected in the field in 2021 and included in the study.
